# Resolution of Large Cervico-Thoracic Syringomyelia Following Treatment of Thyrotoxicosis: A Case Report

**DOI:** 10.7759/cureus.42372

**Published:** 2023-07-24

**Authors:** Claudia L Craven, Sophie Mullins, Laura Pradini-Santos, Laurence D Watkins

**Affiliations:** 1 Neurosurgery, National Hospital for Neurology and Neurosurgery, London, GBR

**Keywords:** hyperthyroidism, thyrotoxicosis, chiari 1, syringomyelia, cerebrospinal fluid

## Abstract

Treatment for a large symptomatic syrinx associated with a Chiari 1 is predominately surgical, via a foramen magnum decompression (FMD), with the aim to normalise cerebrospinal fluid (CSF) movement. Whilst theories of underlying hyperdynamic states in Chiari 1 and Syringomyelia exist, to date there is no effective medical treatment to reverse Syringomyelia.

A 17-year-old female was referred with a seven-month history of gradually progressive impaired temperature sensation in her left upper limb. She had also been concomitantly diagnosed with thyrotoxicosis. Magnetic resonance imaging (MRI) confirmed a Chiari 1 with a large syrinx. The patient preferred to avoid surgery in the first instance. She underwent treatment for her thyrotoxicosis. The eight-month, 20- and 36-month follow-up MRI scans demonstrated a gradual resolution of the Chiari 1 malformation and the syrinx.

Whilst there have been reports of Chiari 1 malformation association with hyperthyroidism, this is the first report describing syrinx resolution following treatment of thyrotoxicosis. Hyperdynamic circulation can result in syrinx formation through various mechanisms. We hypothesise that the treatment of thyrotoxicosis resulted in normalisation of CSF pulse amplitude and subsequent syrinx resolution. Hyperthyroidism evaluation may be explored in studies of CM1 and Syrinx or other CSF disorders.

## Introduction

Natural history of Syringomyelia

Syringomyelia is a cystic cavitation of the spinal cord that is commonly associated with Chiari I malformation (CM1) [1]. The natural history of syringomyelia is known to be highly variable. Patients may be severely symptomatic, with sensory disturbance, pain, and even syringobulbia, whilst others may have a large syrinx and be entirely asymptomatic [1-3].

Management of Syringomyelia

In light of the unpredictable natural history, the management questions that Klekamp et al. raised in 2001 remain relevant today, “Should every patient with Chiari I malformation (and a Syrinx) undergo surgery? Should surgery be postponed until symptoms start to develop?” [3]. An online survey in 2018 revealed that over 80% of surgeons would recommend surgery for asymptomatic patients with CM1 and large syrinxes (8mm) [4]. This is despite evidence that conservative management has been shown to suffice or be equally as effective in the management of idiopathic syringomyelia [5]. A common surgical management strategy for large syrinxes associated with CM1 is a foramen magnum decompression (FMD), with the aim of this surgery to normalise cerebrospinal fluid (CSF) movement [6]. However, FMD is not without risk, and many surgeons will opt for a non-operative approach where possible, taking surveillance strategy in asymptomatic patients [5,7].

Spontaneously resolving syrinxes

Further complicating decision-making is the ever-increasing literature of large syrinxes spontaneously resolving [2]. Herein, we present a case of an extensive and symptomatic syrinx, associated with a CM1, which resolved prior to surgical intervention. What makes this case unique is the presence of hyperthyroidism, and the subsequent reversal of symptoms and radiological signs upon optimal endocrine treatment.

Hypothesis

We hypothesise that this patient had a hyperdynamic CSF pulsatility and that the treatment of this patient’s thyrotoxicosis resulted in normalisation of CSF dynamics, an ultimately syrinx resolution.

## Case presentation

Clinical presentation

We present a case of a 17-year-old female with a large syrinx and CM1. She was referred with a seven-month history of gradually progressive impaired temperature sensation in her left upper limb. She had also been concomitantly diagnosed with thyrotoxicosis. She had a family history, with her father also having a CM1 treated with FMD and ventriculoperitoneal (VP) shunt.

Examination findings

Clinical findings relieved normal power throughout, reduced sensation in left C4-T2 dermatomes in the pinprick and temperature modalities; and absent left biceps and supinator reflexes and reduced left triceps reflex.

Imaging

**Figure 1:**
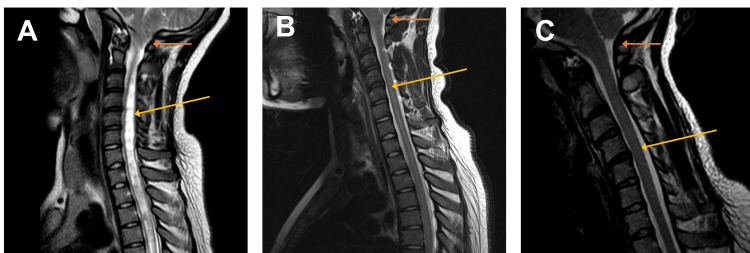
T2-weighted MRI of the cervical spine (sagittal section) (A) Large syringomyelia with extension into the thoracic spinal cord (yellow arrow) and tonsillar herniation (orange arrow) in keeping with Chiari 1 malformation. (B) Improvement of the syrinx (yellow arrow) and improvement of tonsillar descent (orange) at eight months post treatment for thyrotoxicosis. (C) Resolution of the syrinx (yellow arrow) and tonsillar descent (orange) at 20 months post treatment for thyrotoxicosis.

Magnetic resonance imaging (MRI) (Figure 1A) confirmed a diagnosis of CM1 (indicated by orange arrow) with a large syrinx (indicated by yellow arrow) extending into the upper thoracic spine.

Management

At the first clinic appointment, all treatment options were discussed, including surgery, given the size of the syrinx and her symptomatology. However, a conservative surveillance approach was preferred by the patient. Concomitantly, treatment for her thyrotoxicosis was commenced by her endocrinology team. This treatment initially started with 40mg Carbimazole twice daily, the dose of which was titrated down in line with her blood results. Her presenting blood test results were T3 4.6 nmol/L (normal range 1.2-2.8 nmol/L), T4 225 nmol/L (normal range 77-155 nmol/L) and thyroid-stimulating hormone (TSH) 0.1 mU/L (normal range 0.3-4 mU/L), consistent with her disease. Her bloods improved to near normal following treatment at 20 months showed T3 1.2 nmol/L, T4 110nml/L and TSH 5.6mU/L. 

Outcome

The patients eight-month follow-up MRI (Figure 1B) demonstrated some improvement in CM1 (orange arrow) with reduced syrinx size (yellow arrow). Two years later the patient commenced radioiodine treatment. Further imaging another 20 months later demonstrated complete resolution of the syrinx (yellow arrow) and the CM1 (orange arrow) (Figure 1C). Resolution of the syrinx was maintained through a further 36 months of follow-up. Clinically, the abnormal reflexes and the reduced sensation in left C4-T2 also had completely resolved.

## Discussion

We present a case of a large spontaneously resolving syrinx, where resolution coincided with the treatment of thyrotoxicosis. Whilst spontaneous resolution of syringomyelia without surgery is a well-reported phenomenon in the literature, particularly since the widespread use of MRI, its pathophysiology remains unknown. This discussion describes various potential mechanisms through which hyperthyroidism may result in syrinx formation.

Aetiology of syrinx formation and resolution

Aetiologies for syrinx formation include pathologies with increased intracranial pressure (ICP) (such as hydrocephalus, space-occupying lesions, head injury, and veno-occlusive disease), CM1, trauma, multiple sclerosis, spinal tumours and idiopathic aetiology [1]. However, there are very few reported endocrine cases of syrinx formation [8].

The first report of an MRI-verified spontaneous resolution of syringomyelia was in 1990 by Sudo et al. [9]. Since then, several cases (with a mixture of pediatric and adult populations) have been reported in the literature, with the majority being associated with CM1 [2,3,8].

Different hypotheses have been proposed for the spontaneous resolution of syringomyelia. In children, the restoration of normal CSF dynamics and thus correction of the syrinx has been attributed to enlargement of the posterior fossa space with age [10]. In adults, the rupture of arachnoid membranes and fissuring of the syringomyelic cavity into the subarachnoid space is a popular working hypothesis. Arachnoid veils are present in 45%-68% of Chiari malformations associated with syringomyelia, and similarly, the breakdown of these veils can lead to the resolution of the syrinx [11]. In this report, we hypothesise that the treatment of this patient’s thyrotoxicosis resulted in the normalisation of CSF flow dynamics, through cardiac cycle mechanisms.

Cardiac cycle and syrinx formation

Hyperdynamic CSF flow at the craniocervical junction may be a causative factor in the development of syrinx in CM1. Pulsatile CSF movement during a cardiac cycle can contribute to syrinx progression [12]. Furthermore, pulse amplitude (the difference between systolic and diastolic ICP pressures) has been shown to be raised in patients with CMI and syrinx [13].

Oldfield et al. explored the relationship between cardiac systole and syringomyelia with dynamic MRI [14]. The downward movement of spinal CSF and syrinx during systole occurred with a concomitant pulsatile descent of the cerebellar tonsils. The authors hypothesised that this systolic pressure pulse of CSF, when not accommodated by the shift of basal cistern fluid into the spinal CSF compartment, acts as a piston further propelling CSF vertically into the spinal cord and causing progressive syringomyelia [15].

We postulate that this patient had a large CSF pulse amplitude secondary to the hyperdynamic state of thyrotoxicosis, thus developing a syrinx as per Oldfield’s theory. The treatment of the hyperthyroid state thus resulted in the normalisation of CSF dynamics, an ultimate syrinx resolution.

Could hyperthyroidism precipitate a syrinx?

There are few reports in the literature of CSF dynamic disturbance due to hyperthyroidism. One case reported by Herwig et al. describes a 32-year-old woman suffering from Grave’s and new hydrocephalus that then resolved after propranolol and carbimazole [15]. Similarly, other reports of hydrocephalus or idiopathic intracranial hypertension and CM1 have been associated with hyperthyroidism [16-18].

Hyperthyroidism has been linked to an increase in cerebral blood flow and therefore cerebral blood volume (CBV) and ICP, as well as to increased CSF pulsations [19,20]. Moreover, thyroxine, through its sodium transport regulatory role as well as the increase in venous pressure, can further alter CSF dynamics [21]. However, the precise mechanism remains to be elucidated, with altered CSF dynamics and increased CSF production constituting potential roles [21].

In this case study, the increased metabolic turnover could have resulted in (i) the increased production of CSF by the choroid plexus, (ii) increased CBV and ICP, (iii) possible abnormal PA due to hyperdynamic state and ultimately and a CM1 and Syrinx formation. The key element in our case report, however, is the fact the syrinx disappeared with medical treatment of the thyrotoxicosis.

Strengths and limitations

This is a case report, and we acknowledge that association is not equivocal to causality. Nonetheless, we consider this case to be valuable, given the correlation between syrinx resolution and concomitant timely treatment. Furthermore, there is evidence in the literature to support the theory that a hyperdynamic state may have been driving abnormal CSF dynamics and this syrinx formation.

## Conclusions

Hyperdynamic circulation and raised pulse amplitude is a known factors in the development of syrinxes with CM1. We hypothesise that the treatment of this patient’s thyrotoxicosis resulted in the normalisation of CSF flow dynamics. Similar cases of spontaneous syrinx resolution may reveal alternative treatment options and elucidate the pathophysiology of this complex condition.
